# "The Hospital" Medical Book Supplement.— No. V

**Published:** 1908-03-21

**Authors:** 


### "THE HOSPITAL"
### MEDICAL BOOK SUPPLEMENT.—No. V.

CONTENTS.

**Cuttings** ... 659
**Gynæcology and Obstetrics—**
Pelvic Inflammations in the Female ... 660
Rotunda Practical Midwifery ... 660
**Dermatology—**
Light and $x$-Ray Treatment of Skin Diseases ... 660
A New Medical Directory—Forthcoming Publications ... 662
**Hygiene—**
The Care of the Body ... 661
Proceedings of the Conference on the Teaching of Hygiene and Temperance in the Universities and Schools ... 661
Nature's Hygiene and Sanitary Chemistry ... 661
**Materia Medica and Therapeutics—**
Materia Medica, Pharmacy, Pharmacology, and Therapeutics 662
A Manual of Prescribing ... 662

### CUTTINGS.

THE general practitioners' library, to be complete, should contain a well-arranged series of cuttings and excerpts. Everyone, no matter how wide his reading or how catholic his taste, has some subject which is of more than ordinary interest to him; some subject to which he has devoted, or to which he wishes to devote, special attention. The acquisition of monographs and theses dealing with these specialities is costly, and, moreover, leads to a plethora of volumes on the library shelves. The rich man may be able to buy for his library everything, say, that has been written on such a subject as adenoids, but he must be a man of more leisure than falls to the lot of the average practitioner who has the time to read through these numerous acquisitions. To extract the best from what has been written, to gain the quintessence of what has been said, a process of exclusion, weeding out, and careful selection has to be gone through, and for that reason we commend the practice of excerpting.

We have already in a former article laid stress on the importance, to the practitioner who wishes to be up to date and to keep himself abreast of the development of the art of medicine, of subscribing to one or more good weekly journals which give the gist of the most recent work and thought in the several branches of modern medicine. But it is obvious that a collection of monthly or weekly issues of such journals, even if neatly bound and more carefully indexed than they usually are, is cumbrous and unwieldy. Every Friday the practitioner receives his copy, which in his leisure hours during the following week he is able to read and digest. Very possibly he reads everything: more probably he merely glances at many articles which have no direct interest for him to fix his attention on one or two special contributions which are informative, instructive, and, from his particular point of view, really helpful. It is likely that weeks or months afterwards he may recollect some detail or fact, some hint that will serve him in practice, some new line of treatment outlined, some new diagnostic aid described upon which he chanced during his leisurely reading. Then the difficulty often is to find the point searched for. Points are not usually indexed, and the difficulty of laboriously glancing through a year's issues is one which will be readily understood by anyone who has had to surmount it.

There are two ways of overcoming it, and both have their obvious advantages and shortcomings. The one consists in underlining any special point in an article and making a marginal note for future reference. This is the easiest and best way, for example, of dealing with points of special advantage or interest in text-books or in valuable monographs that cannot be subjected to cutting. An extension of this method is excerpting. For this several small note-books are required, in which points as they are met with in the week's reading are entered in alphabetical or subject order. This method obviates the necessity of referring to the original journals or text-books, which, after excerption, may be destroyed or relegated to an upper shelf, where they are available for more minute reference if required. Its objection is, of course, the amount of time and care which is entailed in successfully indexing and transcribing points.

The second method is, for the purposes of the general practitioner, by far preferable. The requisites are a large scrap-book, strongly bound, with half-leaves as inset among the whole pages, a paste-pot and brush, a pair of scissors or a sharp knife, writing materials, and the journals. Whenever a point is noted it is cut out bodily and pasted in the scrap-book and a line of reference added below. Articles which are really helpful and worth preservation are likewise cut out and pasted in on the half-leaves. Such whole articles generally need no reference annotation, since the name and date of the journal appear on the corner of the page torn out. If the practitioner takes an interest in many subjects a number of scrap-books will, of course, be required, and in that case a cross reference index, though not essential, will greatly add to the ultimate usefulness of these "cutting-books." A convenient plan is to tear out the page or article which it is desired to preserve, and to place it in a special drawer or table basket kept for that purpose. At the end of every quarter, or more often if thought fit, these papers can be sorted and the more useful ones pasted into the scrap-book. Very little labour is involved in the task of selection if carried out in this manner, and the huge pile of papers which contain very little of real interest or value to the practising physician can in this way easily be cut down. The scrap-book, in course of time, will contain the cream of information on the special subject with which it deals, and as a handy work of reference its value is incalculable. Such a system of excerption has been systematically used at the Polyclinic, whose reading-room contains a well-arranged series of scrap-books, properly indexed, filled with cuttings from all sources on subjects of interest to the medical practitioner.

### GYNÆCOLOGY AND OBSTETRICS.

Pelvic Inflammations in the Female. By Thos. Wilson, M.D., F.R.C.S., Obstetric Officer to the General Hospital, Birmingham, etc. Pp. 66. (Bristol: J. Wright and Company. Price 3s. 6d. net.)

This is a publication in book form of Dr. Wilson's Ingleby Lectures of 1907 at the Birmingham University. It contains much detailed and valuable statistical information on the subjects in the title, and the discussion of the anatomy, pathology, ætiology, etc., of the pelvic viscera and their inflammatory diseases is exhaustive and scholarly. A full half of the entire volume is given up to this part of the lecture, a proportion which, perhaps intentionally, gives an academic flavour to the essay, but detracts from its practical utility to the general practitioner. So, too, statistics of operations and their results are of interest rather to the consulting gynæcologist, for they do not give much indication to the practitioner as to when expectant treatment of particular conditions and cases should be abandoned. Dr. Wilson, if we judge correctly, is not a great believer in conservative treatment; he lays considerable stress upon the diagnosis of suppuration, and seems to imply that this necessitates operation. There are, however, a good many gynæcologists who hold that pelvic pus is frequently absorbed naturally. This, if true, may be one of the reasons why leucocyte counts have fallen into disfavour as aids to treatment in gynæcological practice, though Dr. Wilson, we notice, regards them as of value. We should have been glad to see rather more stress laid upon the importance of the way in which these cases should be nursed, and of the very strict interpretation to be placed on the term "absolute rest"; for these are details of the utmost consequence in the treatment of pelvic inflammatory troubles. As a summary of modern views on topics of which no medical man can afford to remain ignorant, the lecturer may rest assured that his monograph will be frequently consulted. It is one worthy of the wider circulation which publication in book form will no doubt assure.

Rotunda Practical Midwifery. By E. Hastings Tweedy, M.D., F.R.C.P.I., Master of the Rotunda Hospital, and G. T. Wrench, M.D., late Assistant Master. Oxford Medical Publications, 1908. (Henry Frowde and Hodder and Stoughton. Pp. 464, illustrated. Price 16s. net.)

This book represents the incorporation of the lectures on obstetrics by the present master with the practical teaching of the Rotunda Hospital, and as an authoritative description of the methods in use there will be read with great interest. It is intended to be a practical guide to those who find themselves newly qualified with "considerable theoretical, but a small amount of practical, knowledge," a condition in which it must be admitted most newly qualified men find themselves. We cannot quite agree with the senior author that the examining bodies must be held "solely accountable" for this lack of practical knowledge, because the requirements of practical midwifery in their curricula are so inadequately provided for. It must be admitted that the curricula of most examining bodies have been drawn up in accordance with the amount of clinical material at present available for teaching purposes, and until this material is greatly increased by the provision of large lying-in hospitals, or wards in general hospitals, we cannot see how the curriculum can be widened so as to demand more of the medical student. The practical note which is struck in the first chapter of the book is well maintained throughout, and in all instances treatment is given clearly and minutely described to the exclusion of alternative methods, which are purposely omitted—not because other methods may not be admirable, but because in the author's opinion they are no better than those described. On the management of labour and on antisepsis the chapters are admirable, and planned in such a manner that one feels such methods could be carried out in the humblest of homes as well as in a lying-in hospital. These chapters should do something to dispel the oft-repeated plaint that it is impossible to carry out aseptic midwifery in a poverty-stricken home. The subject of abortion is very fully dealt with, and the indications for and methods of clearing out the uterus are most clearly laid down. We naturally looked for authoritative teaching on this point, and also upon that of antepartum hæmorrhage, and whilst we find the former admirable we confess to being somewhat disappointed in the latter. It is true that the Rotunda method of plugging the vagina is fully dealt with and carries absolute conviction as to its propriety, but we had hoped to see something more definite laid down on the treatment of concealed hæmorrhage. We cannot agree that vaginal Cæsarean section will ever take a place in the armamentarium of the general practitioner. The treatment of eclampsia is excellently dealt with, but we find no mention of the conduct of pregnancy with diabetes, and chorea of pregnancy is dismissed in four lines. The chapters on contracted pelvis and obstetrical operations are highly practical, and those on the infant, which conclude the book, are among the best in it. The illustrations are excellent on the whole, though some are a little crude from an artistic point of view. We note with great pleasure that the operator's hands are always depicted with gloves on, illustrating forcibly the greatest advance perhaps, in midwifery of modern times.

### DERMATOLOGY.

Light and X-Ray Treatment of Skin Diseases. By Malcolm Morris, F.R.C.S.(Ed.) and S. E. Dore, M.D.(Camb.). Pp. 172. Plates xii. (London: Cassell and Company. Price 5s.)

When specialisation in any medical subject depends for its basis upon particular methods of treatment rather than upon anatomical or pathological grounds the door is at once opened for enthusiasm, with the result that the curative effects of each particular line of treatment are apt to be expressed only by superlatives. It is all the more refreshing, therefore, to read such a book as this, in which the authors play the part of unbiassed judges, and in a very broad-minded and level-headed way summarise the *pros* and *cons* of the subject. The book is not full of physical technicalities, elaborate descriptions of apparatus, and so forth. It deals much more with the results of the special forms of treatment than with the actual methods of applying them. In other words, it is not written so much for those who are likely to have charge of the actual application of the various rays as for those who would like to know which form of treatment will probably do most good in any particular case. There is no doubt that Finsen light, *x*-rays, radium, and even high-frequency currents all afford additional means of relieving certain skin diseases; but in the minds of many the extent to which the various treatments are really curative is very vague. Some still look upon such treatment with misgiving, and hesitate to recommend it; others are too enthusiastic, and expect too much from it. A book such as this cannot but be helpful to all who read it, in that it gives so judicial a summary of the results obtained up to the present, pointing out the limitations, the indications,

ıe possible ill-effects, the contra-indications, and the pro-able results of each form of ray treatment for various skin conditions. Lupus vulgaris and rodent ulcer naturally receive the major share of attention; but ringworm, benign and malignant new growth, mycosis fungoides, cheloid, hypertrichosis, naevi, warts, alopecia, sycosis, psoriasis, and many other conditions are dealt with also. If there re two faults to be found with the book they are hese: First, that the authors have not restricted themselves to their own personal experiences throughout; secondly, that they have confined themselves almost exclusively to the results of the application of the various rays without entering very much into the other forms of treatment that might be used concurrently. The result of the first of our objections is that the judicial attitude is lost in many places when the authors introduce the opinions of others; we would much rather have the personal experience of the authors alone throughout the book, without the tinge of undue enthusiasm which creeps in when quotations are made. The result of the second objection is that the reader must consult other authorities, or his own experience, to know whether it is better to trust to rays alone or to use other measures also. The authors admit that the newer therapeutic measures, although a tremendous advance upon any hitherto known for some conditions, are not a panacea by themselves; and yet there is little discussion as to when the rays should be assisted and when not. We are surprised, for example, that there is no mention of the tuberculin treatment of lupus vulgaris concomitantly with the Finsen light. It may be that the authors do not approve of the combination, but still it might well have been discussed. There are many who believe much in the Finsen light, but much more in light treatment assisted by tuberculin.

We can only repeat that we have read it with pleasure, and that it is a very useful addition to the literature upon the subject.

### HYGIENE.

The Care of the Body. By Francis Cavanagh, M.D., Edin. (London: Messrs. Methuen and Co. Pp. 292. Price 7s. 6d. net.)

This book, as its title suggests, is written upon popular lines for the general public. It deals in a very readable manner with the personal hygiene of every-day life. The author points out that modern conditions of life are so different from those of the rural past that it is every day more necessary that one should know the general rules of most healthy living if one is to keep well. This is, perhaps, true in some cases, but we think that the person who lays much conscious stress upon his rules of life, wondering constantly what he can and what he cannot eat, wear, and do, is more pathological than one who lives ordinarily without worrying about these things. The book, therefore, may do harm as well as good: good in the case of those who can make themselves masters of the rules discussed in it; harm in the case of those who, on reading about such things, allow the rules to obtain too great a mastery over them. It is, nevertheless, true that many people live in a less healthy way than they need do, simply because they are not taught the general principles of personal hygiene when they are young. These things should be taught to children by their parents, and in the schools; and as a text-book for teachers the book before us will be admirable. The points dealt with include the physiology and pathology of such things as sleep, baths, exercise, fatigue, massage, clothing, the hair, the teeth, the eyes, lighting and ventilation; the question of drink; food; posture; and general habits. The book ends with a curious chapter in which the author insists that every person should be examined medically once a quarter. We think the tendency of the book is to regard the limits within which the body can remain healthy as too narrow, the result of which must be that some who read it will be liable to become too conscious about their bodies instead of living ordinary healthy lives, the rules of which should all be quite sub-conscious.

Proceedings of the Conference on the Teaching of Hygiene and Temperance in the Universities and Schools of the British Empire. (London: John Bale, Sons and Danielsson. Pp. 129. Price 2s. net.)

The Conference, of which this little volume gives the Proceedings, was held at the Examination Hall, Victoria Embankment, under the chairmanship of Lord Strathcona, Sir John Cockburn, and Sir John Gorst. It was not an annual but a special Conference, called together in order to utilise the visit of many officials and others from the Colonies, in connection with the Colonial Conference, to obtain information as to the steps that had been already taken in the outlying parts of the Empire to introduce this teaching. Two things are quite clear from the evidence collected together—namely, first, that many even of our most outlying Colonies are far ahead of the Mother Country in the teaching of elementary, personal, and general hygiene, and temperance to the younger generation; and, secondly, that not only is such teaching not difficult to carry out, but it is really beneficial and not in any way detrimental to those who are taught. If these sorts of things, rather than religious points, formed the basis for discussion in matters relating to Education Bills, the Mother Country would not be left behind. The secret of the success of the work in these distant Crown Colonies has been twofold: in the first place the teaching has had a direct bearing upon the special needs of the community, and in the second place it has been scientific and not didactic. We think that the book, unpretentious as it is, and uninviting as its title looks, deserves a very wide circulation amongst all branches of the community.

Nature's Hygiene and Sanitary Chemistry. By C. T. Kingzett, F.I.C., F.C.S. Fifth edition. (London: Baillière, Tindall, and Cox. Pp. 527. Price 10s., or 7s. 6d. net.)

This book clearly serves a useful purpose, as is shown by the fact that it has reached its fifth edition since it originally appeared in 1880. It is written rather for the general public than for the medical profession; and although it contains much that is of interest to doctors, the general idea of the book is what one would term popular. The first four chapters deal with the elementary principles of chemistry, such as: the constitution of matter, atomic weights, valency, reactions and simple equations; the composition of the atmosphere; the principles of oxidation, respiration, ventilation, combustion, natural decay, putrefaction, fermentation; the rôle of micro-organisms in these processes; their influence on health. The next two chapters deal in a general way with different varieties of natural waters, sewage purification, and so forth. The succeeding chapters discuss the chemical and germ theories of infectious and contagious diseases, phagocytosis, immunity, and disinfection. The portions of the book in which the author's own researches come most to the fore are those which discuss disinfectants and their standardisation; and the various essential oils; and the book ends with a succinct account of eucalyptus, pine, and camphor forests, and an account of the industries connected with them.

### MATERIA MEDICA AND THERAPEUTICS.

Materia Medica, Pharmacy, Pharmacology, and Therapeutics. By W. Hale White, M.D., F.R.C.P., Physician to, and Lecturer on Medicine at, Guy's Hospital. Pp. 692. (London : J. and A. Churchill. Tenth edition. Price 7s. 6d. net.)

This text-book of materia medica and allied subjects is so well known to all that it is scarcely necessary to do more than state that another edition of it has been called for. So great is the demand for it, especially amongst medical students, that the editions have been following one another with the utmost regularity at intervals of about eighteen months. It is only of recent years that anything like a competitor has stood up against it, and even now it covers a ground which is covered by no other book of the same size. Other books upon materia medica, upon pharmacy, and upon therapeutics there are, and good ones. Those who wish to read of therapeutics only would probably buy a different book; those who wish to read of materia medica, pharmacy, and pharmacology, without troubling about therapeutics, would no doubt prefer the Codex recently issued by the Pharmaceutical Society. Amongst students, however, Hale White's text-book deservedly maintains its popularity, and will continue to do so. So well known a publication can afford to be criticised minutely, but we can only mention one or two matters amongst many that we should like to comment on. Digitalis is one of these. The drug is so important that we cannot help feeling that the student should be told everything about it very fully, even though to make space for details the accounts of some of the rarer drugs had to be curtailed. We should like to have seen it stated that digitalis chiefly causes vomiting when it is given along with other drugs, such as nux vomica, or caffeine, and the like; the student should be able to learn from his text-book that vomiting comparatively seldom results if tincture of digitalis is given in water by itself. Again, considerable stress is laid upon that bugbear, the cumulative action of digitalis, and yet no mention is made of the possibility that the tincture of digitalis that is being administered may contain little or no active principle at all; we have discussed this matter in a former issue, when we spoke of the recent experiments that have been carried out at Cambridge upon the subject, and of the more or less successful attempts that have been made to standardise the tincture. No stress is laid upon the necessity for giving full doses when once it has been decided that digitalis is clearly indicated in the case before one; doses of 15 minims or more three times a day will often produce marked improvement within a week, when 10 minim doses would do very little good at all, but the student is not told this in the book. We have picked out digitalis for criticism because it is such an important drug; and we think that much more should be said about it, notwithstanding the difficulties as to space. It is, unfortunately, always the way that when one really wants to find out fresh things about subjects of which one already knows a little, no single book can, as a rule, supply just the information one requires. To give one other instance in the present case, we are delighted to see the sections devoted to therapeutic sera and to vaccines considerably enlarged in the present edition; but when we look up the vaccine treatment of acne and wish to learn about the quantities to be injected at a time, all we can find is that "a minute dose is injected subcutaneously." We are not told that this "minute dose" usually contains the products of 250,000,000 staphylococci; we are not even told how the dosage is measured. It is for reasons such as these that the qualified practitioner will find less use for the bool than the unqualified student. It is amongst medical student that the volume will continue to find its greatest sale, an for them there is no better book upon these subjects.

A Manual of Prescribing. By C. R. Marshall, M.D Professor of Materia Medica and Therapeutics in tl University of St. Andrews. (London: J. and A Churchill. Pp. 373. Price 5s. net.)

Professor Marshall's manual is propounded fc students and practitioners of medicine: of the former w will venture to say few will read it, though this by no mear proves that it is unworthy to be read; of the latter a grea many of those who of necessity do their own dispensin might study its pages to great advantage. Correct an elegant prescribing can only be founded on an appreciatio of its practical results obtained by actual experience. Mos prescribers who do not dispense are content that their com positions shall contain no incompatibles and shall not b too nasty; beyond that they leave to the ingenuity of th dispenser many difficulties which it is his lot to surmoun as best he may. The better way is that indicated in the page of this book. Lament has frequently been made that th modern medical student learns little of the art of prescrib ing from his teachers, and emerges into practice in a deplorable state of ignorance in this respect. The charge is too often well founded, but we should expect to find many exceptions among the pupils of Professor Marshall at St. Andrews University. We fear it is too much to expect of those elsewhere that they should absorb his teaching through his book, for they have so many other subjects to master. Nevertheless we shall venture to recommend it to all members of the profession, both senior and junior.

**Forthcoming Publications.**

